# Evolution of reproductive isolation as a by-product of divergent life-history evolution in laboratory populations of *Drosophila melanogaster*

**DOI:** 10.1002/ece3.413

**Published:** 2012-11-19

**Authors:** Shampa M Ghosh, Amitabh Joshi

**Affiliations:** 1 Department of Zoology, Michigan State UniversityEast Lansing, MI, 48824, USA; 2 Evolutionary Biology Laboratory, Evolutionary and Organismal Biology Unit, Jawaharlal Nehru Centre for Advanced Scientific ResearchJakkur PO, Bangalore, 560064, India

**Keywords:** Body size, development time, divergent selection, laboratory selection, mate choice, postcopulatory prezygotic isolation, premating isolation, reproductive isolation, sexual conflict, sexual selection

## Abstract

We show that two complementary asymmetric isolating mechanisms, likely mediated by divergence in body size, underlie the evolution of incipient reproductive isolation between a set of *Drosophila melanogaster* populations selected for rapid development and their ancestral controls. Selection has led to great reduction in body size in the fast developing lines. Small males belonging to fast developing lines obtain few matings with large control females, both in presence and absence of large control line males, giving rise to unidirectional, premating isolation caused by sexual selection. Conversely, small selected line females suffer greatly increased mortality following mating with large control males, causing unidirectional postcopulatory prezygotic isolation. We discuss preliminary evidence for evolution of reduced male harm caused to females upon mating in the fast developing lines, and speculate that the females from these lines have coevolved reduced resistance to male harm such that they can no longer resist the harm caused by males from control lines. This potentially implicates differing levels of sexual conflict in creating reproductive barrier between the selected line females and the control males. We also show that a large difference in development time is not sufficient to cause postzygotic incompatibilities in the two sets of populations reaffirming the belief that prezygotic isolation can evolve much earlier than postzygotic isolation.

## Introduction

How new species are formed remains one major question in evolutionary biology. The focus of speciation research in recent years has gradually shifted from broad geography-based models of sympatry versus allopatry toward understanding the mechanisms that give rise to reproductive isolation (RI) potentially resulting in speciation (Rice and Hostert 1993; Schluter 2001; Coyne and Orr 2004; Rundle and Nosil 2005; Butlin and Ritchie 2009; Fry 2009; Butlin et al. 2012). Experimental evolution and laboratory selection approaches are particularly useful for understanding the mechanistic bases of evolutionary processes and such methods are being employed increasingly to study the evolution of RI (Reviewed in Rice and Hostert 1993; Ritchie 2007). While several laboratory studies have shown that partial RI can evolve as a correlated response to divergent selection on behaviors (Del Solar 1966; Hurd and Eisenberg 1975; Markow 1981; Lofdahl et al. 1992) or life-history traits (Miyatake and Shimizu 1999), or adaptation to different environments (Kilias et al. 1980; Rice and Salt 1988; Dodd 1989; Boake et al. 2002; Rundle et al. 2005; Vines and Schluter 2006; Dettman et al. 2007, 2008), studies providing evidence of the mechanisms underlying the correlated evolution of RI are meager (reviewed in Coyne and Orr 2004; Fry 2009). In the melonfly *Bactrocera cucurbitae*, selection for slow and fast preadult development led to changes in circadian clock period that, in turn, led to the evolution of RI due to changed phase of the circadian mating rhythm (Miyatake and Shimizu 1999). Divergent adaptation to nutritional (Rundle et al. 2005: *Drosophila serrata*) aspects of different environments can directly result in evolutionary shifts in display and reception of visual or chemical signals involved in mate recognition/choice. Such results provide evidence for the involvement of “classic” sexual selection in mediating RI. In yeast and *Neurospora*, adaptation to different environments has been shown to result in RI via genetic incompatibilities (Dettman et al. 2007, 2008).

In this study, we focus on the possible role of mating success and sexual conflict, both likely mediated by divergent body size evolution, in creating reproductive barriers between populations of *Drosophila melanogaster* selected for rapid preadult development and their ancestral controls. Sexual selection has long been thought to be an important driver of speciation, because it directly acts on traits related to mate recognition and reproductive success, and species typically show considerable divergence in such traits (West-Eberherd 1983; Panhuis et al. 2001; Ritchie 2007). The degree and precise form of sexual selection can differ among populations undergoing divergent adaptation, and this has been thought to cause reproductive traits to diverge between populations, potentially leading to the formation of reproductive barriers (Panhuis et al. 2001; Arnqvist and Rowe 2005; Rundle and Nosil 2005; Ritchie 2007). However, most evidence in support of the role of sexual selection in speciation comes from comparative data, and clear empirical support for this view is largely lacking (Panhuis et al. 2001; Kraaijeveld et al. 2011; but see Boughman 2001). It is also increasingly realized that divergent sexual selection, in the sense of phenotypic variation being correlated with differential mating success, can result in myriad direct and indirect ways from divergent ecological adaptation (Ritchie 2007; Maan and Seehausen 2011).

Another phenomenon related to sexual selection is sexual conflict, which arises when traits that increase fitness in one sex simultaneously impose fitness costs on the opposite sex (Chapman et al. 2003). Such conflicts can give rise to sexually antagonistic coevolution, where adaptive change in one sex leads to counter-adaptation in the other (Chapman et al. 2003). Sexual conflict can also bring about rapid changes in reproductive traits and has been thought to play a role in generating RI between diverging populations (Parker and Partridge 1998; Rice 1998; Arnqvist et al. 2000; Gavrilets 2000; Gavrilets et al. 2001). Again, as in the case of sexual selection, there is a paucity of clear empirical data linking sexual conflict and RI (Panhuis et al. 2001; Gavrilets and Hayashi 2005; Ritchie 2007; Kraaijeveld et al. 2011; Maan and Seehausen 2011). Although laboratory selection has been fruitfully deployed to study the evolutionary consequences of sexual selection and sexual conflict in general (Rice 1996; Blows 2002; Rundle et al. 2005; Prasad et al. 2007; Ritchie 2007; Morrow et al. 2008; Edward et al. 2010; García-González 2011), empirical evidence for links between sexual selection/conflict and evolution of RI is inconsistent. Increasing the level of sexual conflict led to the evolution of RI in some studies (Martin and Hosken 2003; Hosken et al. 2009), but not in others (Wigby and Chapman 2006; Bacigalupe et al. 2007; Gay et al. 2009).

Body size, an important life-history trait ontogenetically linking the preadult and adult stages in holometabolous insects, is strongly correlated with preadult development time in *D. melanogaster* (Chippindale et al. 1997; Prasad and Joshi 2003). Selection for rapid development in *D. melanogaster* has repeatedly been shown to result in the correlated evolution of smaller body size (Zwaan et al. 1995; Nunney 1996; Chippindale et al. 1997; Prasad et al. 2000). Moreover, body size is also known to play a significant role in sexual selection and sexual conflict in *Drosophila*. Large females are generally more fecund (Stearns 1992; Roff 2002) and are often preferred by males (Andersson 1994; Byrne and Rice 2006). Bigger size typically confers greater competitive ability in male-male competition in *D. melanogaster* (Partridge and Farquhar 1983; Partridge et al. 1987a,b; Markow 1988; Markow and Ricker 1992), and large males are also often preferred by female flies (Ewing 1961; Markow 1986; Partridge et al. 1987a; Pitnick 1991). However, the preference for larger males can also give rise to sexual conflict because mating with large males reduces female lifespan and egg-production rates (Pitnick and García-González 2002; Friberg and Arnqvist 2003; Taylor et al. 2008). Given that both development time and body size in *Drosophila* can evolve in response to a variety of selection pressures in both laboratory and natural habitat (reviewed by Prasad and Joshi 2003), we focus here on the influence of development time and body size evolution on reproductive traits in laboratory populations of *D. melanogaster* to examine whether mating success and sexual conflict may be mediating RI in this system.

**Figure 1:**
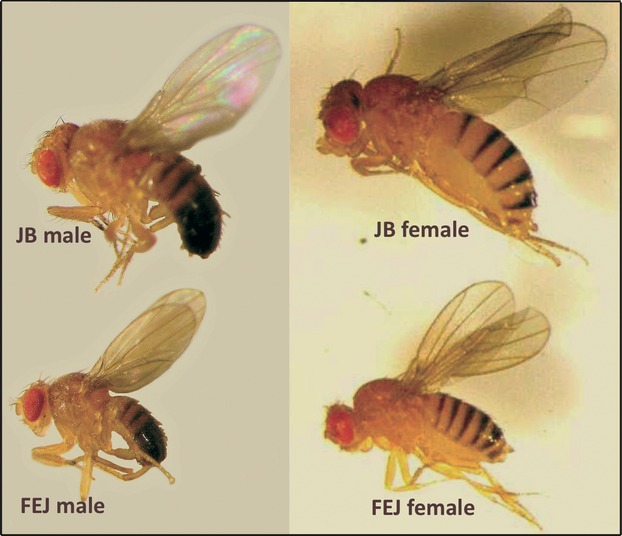
Laboratory populations of *Drosophila melanogaster* selected for rapid preadult development and early reproduction for 370 generations have undergone 25% reduction in development time and >50% reduction in body size compared to their ancestral controls. Flies from control populations (Joshi Baseline [JB]) shown on top, and those from selected populations (Faster reproducing, Early reproducing, JB-derived [FEJ]) shown below.

We studied a set of four laboratory populations of *D. melanogaster* that have been selected for rapid development for over 300 generations, and have also evolved reduced body size relative to the four ancestral control populations (Fig. 1; Prasad et al. 2000, 2001; Ghosh-Modak 2009). The selected populations have also evolved reduced lifespan and fecundity, preadult larval competitive ability, changes in larval behavioral traits, and decreased resistance to biotic and abiotic stresses during both larval and adult stages (Prasad et al. 2000, 2001; Prasad 2004; Shakarad et al. 2005; Ghosh-Modak 2009; Ghosh-Modak et al. 2009). We tested for RI between the selected populations and their ancestral controls, and found evidence for two complementary asymmetric pre and postmating barriers to effective reproduction between selected and control populations. We found no evidence for any direct effect of the large life-history divergence between selected and control populations on postzygotic RI through genetic incompatibility resulting in hybrid breakdown. We discuss our results in the light of sexual selection and possible sexual conflict in these populations, and show how the likely mechanism of the evolution of RI in this study underscores the subtlety with which natural selection and sexual selection can interact to yield isolation.

## Materials and Methods

### Experimental populations

We used eight laboratory populations of *D. melanogaster*: four selected for rapid development and early reproduction, (FEJ_1–4_: Fast development, Early reproduction, derived from JB, first described by Prasad et al. 2000), and their four matched ancestral control populations (JB_1–4_: Joshi Baseline, first described by Sheeba et al. 1998). All populations were maintained on discrete generations at ∼25°C, ∼90% relative humidity and constant light on banana-jaggery food. In both the JBs and FEJs, larvae were reared in glass vials (2.4 cm dia × 9 cm ht) with 6 mL food at a density of 60–80 larvae per vial, whereas eclosed adults were collected into Plexiglas cages (25 × 20 × 15 cm^3^) with abundant food, at breeding population sizes of 1500–1800 flies. The JBs were on a 3-week discrete generation cycle, and all eclosing adults were part of the breeding population. In the FEJs, only the earliest 20–25% of eclosing flies in each vial were collected into cages to form the breeding population, and eggs for initiating the next generation were collected on the third day posteclosion. To equalize breeding adult numbers, many more vials with eggs were set up in case of the FEJs. Thus, the FEJs were under strong primary selection to complete egg-to-adult development rapidly, and under secondary selection to be relatively fecund on day 3 of adult life. As each FEJ population was derived from one JB population, selected and control populations bearing identical numerical subscripts were more related to each other than to other populations in the same selection regime. Consequently, control and selected populations with identical subscripts were treated as blocks in the statistical analyses. At the time of this study, the FEJs had undergone about 370 generations of selection, and showed considerable evolutionary reductions in development time (∼25%), dry weight/size (∼50%; Fig. 1) and general level of activity (Prasad et al. 2000, 2001; Ghosh-Modak 2009).

### Collection of flies for assays

Prior to assays, all eight populations were reared under a common (control JB type) regime for one complete generation in order to ameliorate nongenetic parental effects, and the progeny of these flies (referred to as “standardized” flies) were used for all experiments described below. For the mating assays, 3-day-old virgin standardized flies were used.

#### Individual mate choice and mating latency

Separate male and female individual choice assays were performed in vials, where each individual was provided with two mating partners of the other sex, one each from the selected and control populations. Ten such vials were set up for each combination of replicate population and selection regime. The identity (selection regime) of the mating partner for the first copulation was noted for each vial, and only copulation events lasting for more than 3 min were considered viable matings. The ratio of homogamic to heterogamic matings was calculated across 10 replicate vials for each combination of replicate population and selection line. The data were subjected to replicated *G*-tests for goodness of fit (Sokal and Rohlf 1998; McDonald 2008), permitting both block-wise and overall testing of the null hypothesis of the random-mating expectation, that is a 1:1 ratio of homogamic to heterogamic matings. In addition, the duration between the introduction of the flies into the vial till the first mating (mating latency) was also recorded. This is a fairly standard design for “mating-choice” assays (Jiggins et al. 2001; Dukas 2005; Westerman et al. 2012) and we therefore label it as such, although we recognize that in all such assays the outcome could be the result of either mate choice by the sole representative of a gender in the vial or of superior ability to compete by one of two types of individuals of the other gender.

#### Mating assay in groups

Forty flies – 10 males and 10 females each from a matched replicate pair of JB and FEJ populations – were introduced together in a glass Petri dish of 17-cm diameter containing a thin layer of food. The number of copulating pairs of each of the four possible mating combinations (JB_*i*_♀ × FEJ_*i*_♂, FEJ_*i*_♀ × JB_*i*_♂, JB_*i*_♀ × JB_*i*_♂, FEJ_*i*_♀ × FEJ_*i*_♂) in an observation period of 1-h was recorded. The size difference between JB and FEJ flies was large enough to determine the mating combination without having to remove the copulating pairs from the Petri dish. The assay was replicated three times for each block, with a different set of 40 flies. The number of copulating pairs for each mating combination was noted for each run. Replicated *G*-tests for goodness of fit testing the null hypothesis of random-mating expectation of a 1:1:1:1 ratio were performed.

#### Female mortality rate with varying male density

Reciprocal crosses at three different male densities (1♀: 1♂; 1♀: 5♂; 1♀: 10♂) were set up in vials containing food using virgin JB and FEJ flies from the same replicate population. The following crosses were set up: (1) JB_*i*_♀ × FEJ_*i*_♂ (2) FEJ_*i*_♀ × JB_*i*_♂ (3) JB_*i*_♀ × JB_*i*_♂, and (4) FEJ_*i*_♀ × FEJ_*i*_♂. For each combination of cross, replicate population and male density, 10 replicate vials were set up, yielding a total of 480 vials for the assay. Females were continuously housed with the specified number of males, and female mortality was noted at 8-h intervals from the time of set up till 328-h after set up. Cumulative mortality of females for each combination of cross × male density at two time points, namely 80- and 328-h was arcsine-square-root transformed and subjected to separate mixed-model analyses of variance (ANOVA) with cross and male density being treated as fixed factors crossed with random blocks. Post-hoc comparisons were carried out using Tukey's HSD test.

#### Hybrid survivorship and development time

To test for postzygotic RI, egg-to-adult viability was assayed for F_1_ and F_2_ progeny of the crosses JB_*i*_♀ × FEJ_*i*_♂, FEJ_*i*_♀ × JB_*i*_♂, JB_*i*_♀ × JB_*i*_♂, and FEJ_*i*_♀ × FEJ_*i*_♂ (*i* = 1–4). The crosses were performed in population cages and progeny survivorship assays were performed in vials at a density of 30 eggs per vial and 10 such vials were used for each combination of cross, replicate population, and generation (F_1_ or F_2_). Vial survivorship values were arcsine-square-root transformed and averaged across vials to obtain population means.

Egg-to-adult development time of the F_1_ progeny was tested at a density of 30 eggs per vial, using 10 replicate vials for each replicate population and cross. Vials were monitored closely and once eclosion began the vials were checked regularly at 2-h intervals and freshly eclosed flies were removed from vials. The number of eclosing adults was recorded. These observations were continued until no new flies eclosed for two consecutive days in any of the vials. From these data, the mean development time of F_1_ flies was calculated. F_1_ development time was averaged across vials to obtain population means.

The replicate population means for each trait (F_1_ development time, F_1_ and F_2_ survivorship) were subjected to separate two-way ANOVAs treating cross (four levels) as a fixed factor crossed with random blocks.

#### F_1_ fecundity

Fecundity of unyeasted F_1_ flies from all four types of crosses was assayed at two different ages, corresponding to the age of egg collection under FEJ and JB maintenance regimes. Thus, daily fecundity per female was assayed for day 2, 3, and 4 (corresponding to the FEJ egg collection) and also day 10, 11, and 12 (corresponding to the JB egg collection). Flies were collected from the F_1_ cages and were placed as pairs in vials containing food. Twenty such vials were set up for each cross × age × block combination using a cohort of 2-day-old flies. The fly-pairs were transferred into a fresh vial every 24 h and the eggs laid were counted for 3 consecutive days and averaged. The fecundity was assayed for day 10, 11, and 12 posteclosion using a different cohort of flies that were collected from the population cage on day 10. Mean fecundity was pooled across ages and averaged across vials and subjected to a two-way ANOVA treating cross (four levels) as a fixed factor crossed with random blocks. All statistical analyses were implemented using STATISTICA for Windows (StatSoft, Inc. 1995).

## Results

### Mate choice assays

**Figure 2:**
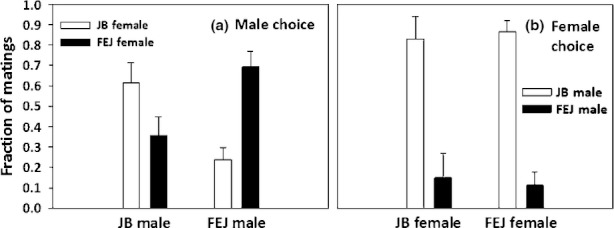
Mean fraction of matings (averaged over four replicate populations and 10 vials) in the (a) male and (b) female choice assays. The error bars represent standard errors across four replicate population means.

In the individual mate choice assay, the ratio of homogamic to heterogamic matings was tested for deviations from the 1:1 null expectation separately for each of the four combinations of selection regime and sex. Three of the four combinations showed a significant deviation from the null hypothesis of random mating, and the fourth was close to significance (Table 1). FEJ males mated significantly more often with FEJ than JB females, when given a choice (Fig. 2a, Table 1). On the other hand, JB females mated significantly more often with JB than FEJ males, when given a choice (Fig. 2b, Table 1). In both cases, the deviation from a 1:1 ratio was significant overall, and there was no heterogeneity among blocks (Table 1). In the case of FEJ females, when given a choice, the overall trend was of significantly greater matings with JB males, but there was also significant heterogeneity among blocks (Fig. 2b, Table 1), and only blocks 1, 2, and 3 showed a significant deviation from the 1:1 expectation (analysis not shown). JB males, when given a choice, mated more often with JB females (Fig. 2a), but the difference was consistently not significant across blocks (Table 1).

**Table 1:** Results of the replicated *G*-test for the individual mate-choice assay

Mate-choice combination	Total *G*	df	*P-*level	Pooled *G*	df	*P-*level	Heterogeneity *G*	df	*P-*level
JB♂ × JB♀, FEJ♀	8.381	4	0.079	3.145	1	0.076	5.236	3	0.155
FEJ♂ × JB♀, FEJ♀	47.564	4	<0.001	44.764	1	<0.001	2.799	3	0.424
JB♀ × JB♂, FEJ♂	12.002	4	<0.001	10.034	1	<0.001	1.969	3	0.579
FEJ♀ × JB♂, FEJ♂	31.692	4	<0.001	20.578	1	<0.001	11.114	3	0.011

FEJ, Faster reproducing, Early reproducing, JB-derived; JB, Joshi Baseline.

**Figure 3:**
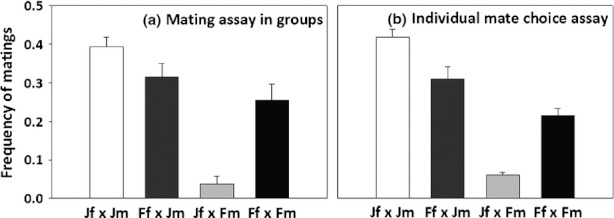
Mean proportion of four types of matings (two homogamic, two heterogamic), averaged over the four replicate populations in (a) the mating assay in groups, and (b) the four individual mate-choice experiments (two male-choices and two female-choices). The error bars represent standard errors across four replicate population means.

In the mating assay in groups, copulation between JB females and FEJ males was extremely rare, only 3.7% of all the matings observed (Fig. 3a). The data for each block were subjected to a *G*-test to examine whether there was significant deviation from a random-mating ratio of 1:1:1:1. Blocks 1, 2, and 4 showed a significant deviation from 1:1:1:1, although block 3 also showed a relatively low frequency of JB♀ × FEJ♂ matings, although nonsignificant (Table 2). There was no significant heterogeneity among within-block replicates in any block (Table 2). When data for each block were pooled across replicate runs, all the four blocks showed significant deviations from 1:1:1:1 ratio as evident from the pooled *G* (Table 2). In the next step of the analysis, the pooled data for all blocks were subjected to replicated *G*-test with the four blocks being treated as replicates. Total *G* and pooled *G* both deviated significantly from the random-mating ratio, whereas no significant heterogeneity was found across blocks (Table 2), indicating that the overall data did show a significant deviation from 1:1:1:1.

**Table 2:** Results of the replicated *G*-test for the group mating assay

	Total *G*	df	*P-*level	Pooled *G*	df	*P-*level	Heterogeneity *G*	df	*P-*level
JB_1_♂, FEJ_1_♂ × JB_1_♀, FEJ_1_♀	25.858	9	0.002	22.619	3	<0.001	3.239	6	0.778
JB_2_♂, FEJ_2_♂ × JB_2_♀, FEJ_2_♀	35.48	9	<0.001	31.787	3	<0.001	3.693	6	0.718
JB_3_♂, FEJ_3_♂ × JB_3_♀, FEJ_3_♀	12.505	9	0.186	9.589	3	0.002	2.916	6	0.819
JB_4_♂, FEJ_4_♂ × JB_4_♀, FEJ_4_♀	28.132	9	<0.001	27.045	3	<0.001	1.087	6	0.982
Pooled	91.04	12	<0.001	75.753	3	<0.001	15.287	9	0.083

FEJ, Faster reproducing, Early reproducing, JB-derived; JB, Joshi Baseline.

**Figure 4:**
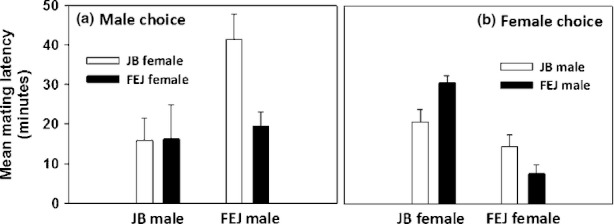
Mean mating latency in (a) male, and (b) female choice assays. The error bars represent standard errors across four replicate population means.

Interestingly, when the data from male and female choice assays were pooled and the frequency of the four mating combinations were calculated, it was very similar to the overall ratios observed in the mating assay in groups (Fig. 3a,b), indicating robustness of the results across the two kinds of assay. In both the individual mate choice and the group mating assays, there was a similar, marked asymmetry in the pattern of heterogamic matings. FEJ males rarely mated with JB females, whereas the other heterogamic mating, that of JB males with FEJ females, did occur at a considerable frequency (Fig. 3a,b). Thus, regardless of whether it is the female or the male that has a choice, or a mixed situation of both sexes having a choice in the mating assay in groups, FEJ males are rarely able to mate with the much larger JB females. Not surprisingly, the mating latency results mirrored the pattern of the mate choice results, with the latencies for FEJ♀ × JB♂ being the highest in both male and female choice assays (Fig. 4a,b).

### Female mortality rate with varying male density

**Figure 5:**
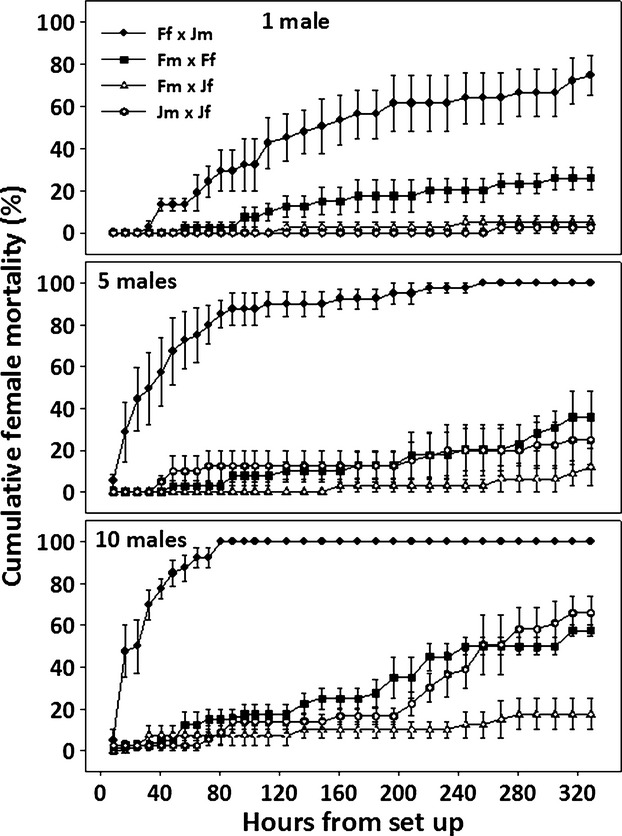
Mean cumulative postcopulation female mortality plotted across time. The error bars represent standard errors across four replicate population means at each time point.

In all the four crosses, cumulative female mortality over time increased with number of male partners, although the effect was marginal in JB♀ × FEJ♂ (Fig. 5). In general, cumulative female mortality increased fastest in FEJ♀ × JB♂, and this cross also showed the greatest sensitivity of female mortality to increasing male density: all FEJ females housed with JB males in 1:10 sex ratio died within 80 h (Fig. 5). ANOVA on cumulative mortality at both 80- and 328-h revealed significant effects of cross, male density and the cross × male density interaction (Table 3). Post-hoc comparisons using Tukey's HSD test showed that cumulative female mortality at 80-h was significantly higher (*P* < 0.05) for FEJ♀ × JB♂ cross than that for the remaining three crosses, and cumulative female mortality of the FEJ♀ × JB♂ cross was significantly less at 1:1 sex ratio than at either 1:5 or 1:10. At 328-h, the general pattern of cumulative female mortality was similar. Cumulative female mortality for FEJ♀ × JB♂ cross was significantly higher than the other crosses, and the cumulative mortality at 1:10 and 1:5 sex ratios for this cross was significantly higher than that at 1:1 sexratio. Moreover, at 328-h, the JB♀ × FEJ♂ cross showed significantly lower cumulative mortality than that observed in FEJ♀ × FEJ♂ cross, a difference not apparent at 80 h (Fig. 5). Thus, the FEJ♀ × JB♂ cross resulted in highest female mortality rate among all the four crosses that was not observed for the other hybrid cross, that is JB♀ × FEJ♂.

**Table 3:** Summary of results of ANOVA done on mean cumulative female mortality 80 and 328 h postcopulation

	50 h				328 h		
		
Source	df	MS	*F*	*P-*level	MS	*F*	*P-*level
Cross	3	2.94626	66.61029	<0.001	2.93176	130.2593	<0.001
No. of males	2	0.93421	10.25178	0.012	0.74833	35.1553	<0.001
Cross × no. of males	6	0.10593	2.68221	0.049	0.18963	4.8722	0.004

### F_1_ and F_2_ survivorship and F_1_ development time

**Figure 6:**
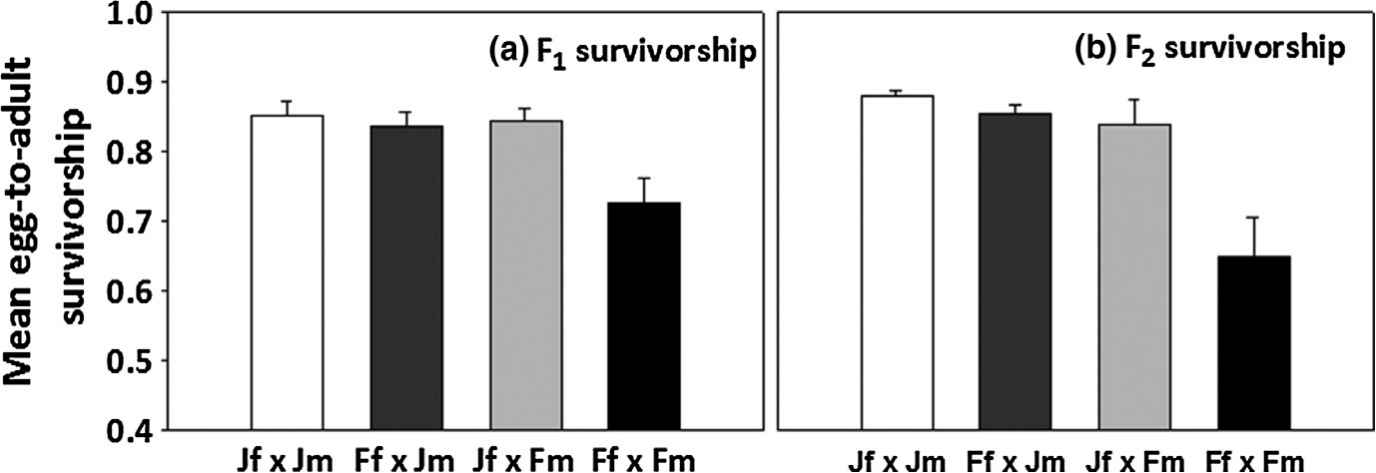
Mean (±SE) egg-to-adult survivorship of the (a) F_1_, and (b) F_2_ progeny.

**Figure 7:**
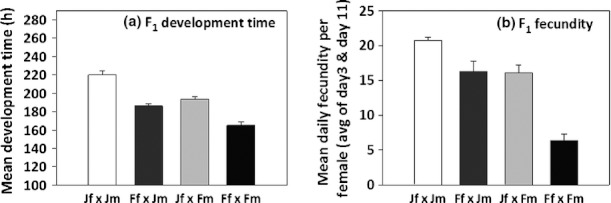
(a) Mean (±SE) egg-to-adult development time of the F_1_ progeny. (b) mean (±SE) number of eggs laid by F_1_ females averaged over day 3 and 11 posteclosion.

The pattern of egg-to-adult survivorship of F_1_ and F_2_ progeny from parental and hybrid crosses was very similar (Fig. 6a,b). In both generations, ANOVA showed a significant effect of cross on survivorship (F_1_: *F*_3,9_ = 11.26, *P* = 0.002; F_2_: *F*_3,9_ = 7.29, *P* = 0.009), and multiple comparisons revealed that the only significant pair-wise differences were those between the progeny of the FEJ♀ × FEJ♂ cross and the progeny of the other three crosses. In contrast to survivorship (Fig. 7a), the F_1_ hybrids showed development time intermediate to that of the parental FEJs and JBs (Fig. 7a). ANOVA revealed a significant main effect of cross (*F*_3,9_ = 202.59, *P* < 0.0001), and multiple comparisons showed that the mean development time of the F_1_ hybrids was significantly different from both the parental types, but the development time of the reciprocal hybrids did not differ significantly from each other.

### F_1_ fecundity

Mean fecundity pooled over day 3 and day 11 posteclosion was highest in the JBs, lower and similar in the two hybrids, and the least in the FEJs (Fig. 7b). The ANOVA revealed a significant effect of cross (*F*_3,9_ = 40.15, *P* < 0.0001), but multiple comparisons showed that the only significant (*P* < 0.05) differences were those between the fecundity of the progeny of FEJ♀ × FEJ♂ cross and the other three crosses.

## Discussion

As evident from the results of the female choice (Figs. 2b, 3b) and the group mating assays (Fig. 3a), the fast developing and small FEJ males obtain very few matings with either JB or FEJ females in presence of the JB males. This is not surprising given the well-known disadvantage of small body size in male-male competition for matings in *Drosophila* (Partridge and Farquhar 1983; Partridge et al. 1987a,b; Markow 1988; Markow and Ricker 1992). More interestingly, in absence of the male-male competition, FEJ males mated three times more often with FEJ females than with JB females (Figs. 2a, 3a). This could be due to two reasons – JB females might exercise a choice against FEJ males; and/or FEJ males show a preference for FEJ females over JB females. FEJ males were observed to court JB females in almost all the cases (although courtship was not quantified), but JB females were often seen to resist mating attempts by the FEJ males, which suggests the choice might be exercised by the JB females. Female *Drosophila* are known to preferentially mate with larger males (Ewing 1961; Markow 1986; Partridge et al. 1987a; Pitnick 1991), but the causal mechanism is not clearly known (Partridge 1988). It is possible that JB females avoid mating with the small FEJ males because of some innate size preference. Alternatively, FEJ males might be less attractive to JB females due to some other reason, such as differences in courtship song or pheromonal cues, or simply because they are not vigorous and active compared to JB males. It could also be that the small FEJ males are just not able to deal easily with mounting and copulating with the much larger JB females (e.g., see Maynard Smith 1956). With the present data, we cannot distinguish among these various possibilities, but the data clearly suggest the evolution of premating RI between FEJ males and JB females, driven by some form of sexual selection in the broad sense. Evolution of premating RI between FEJ males and JB females is supported by the finding that the longest mating latency is observed in this type of cross (Fig. 4a,b).

There is no possibility in our populations of the kind of circadian clock mediated RI seen in the fast and slow developing *B. cucurbitae* populations of Miyatake and Shimizu (1999). Such mating phase dependent isolation would be expected to be symmetric across both types of heterogamic mating. Moreover, although there is evidence for some effect of the eclosion circadian rhythm on development time in *D. melanogaster* populations sharing ancestry with those used in this study (Paranjpe et al. 2005), there is no clear circadian rhythm in mating exhibited by our populations, as they are housed under constant light (V. K. Sharma, pers. comm, 2012).

While there is no impediment to the other type of heterogamic mating between JB males and FEJ females, the results of the postcopulation female mortality assay (Fig. 5) indicate the existence of postcopulatory RI between JB males and FEJ females due to the high mortality suffered by the FEJ females in this type of cross. There is preliminary evidence suggestive of this barrier being driven by differing levels of sexual conflict between the JB and FEJ populations. Female flies are known to show reduced lifetime fitness as a consequence of mating (Partridge et al. 1987c; Partridge and Fowler 1990), mediated by harmful effects of both male courtship and male accessory gland proteins (Acps) transferred to the female's body during mating (Chapman et al. 1995; Wigby and Chapman 2005), and this fitness cost to females is known to rise with increased body size of their mating partners (Pitnick and García-González 2002; Friberg and Arnqvist 2003). The high mortality rate of FEJ females after mating with JB males (Fig. 5) could be due to various reasons. First, it is possible that the amount/composition of seminal fluid proteins transferred by JB males is more toxic than what the FEJ females have evolved to deal with over the course of a few hundred generations of laboratory evolution. Microarray data from whole adult flies show that many of the accessory gland proteins (Acps), including Acp76A, Acp36DE, Acp98AB, Acp26Aa, Acp76A, Acp53C14a, Acp36DE, Acp70A, Acp95EF, Acp53C14c, and Acp63F have undergone 1.5–7-fold downregulation in FEJ males compared to JB males (Satish 2010; K. M. Satish, P. Dey, and A. Joshi, unpubl. data). We speculate that the FEJ males have undergone an evolutionary reduction in Acp production, perhaps as a correlate of reduced body size, and female resistance to the toxic effects of Acps has also consequently reduced over the 370 generations of laboratory selection for rapid development. In the FEJs, conservation of energy reserves is important to fitness because the flies have very low lipid levels at eclosion due to the reduced third larval instar duration; however, they need to be relatively fecund on day 3 posteclosion because of the selection regime (Prasad 2004). In general, the FEJs appear to have evolved a syndrome of reduced energy expenditure, relative to the JB controls (Prasad et al. 2001). This might have led to a reduced energy expenditure for Acp production in males, and female resistance in females in the FEJs, resulting in FEJ females paying a fatal cost upon mating with the large JB males. In *D. melanogaster*, variation in female resistance to male harm was documented by Linder and Rice (2005), and female resistance evolved in experimental evolution studies manipulating the levels of sexual conflict (Holland and Rice 1999; Wigby and Chapman 2004; Lew et al. 2006). Reproductive traits in *Drosophila* including Acp levels are known to undergo rapid evolutionary change (Swanson et al. 2001; Swanson and Vacquier 2002; Wolfner 2002; Begun and Lindfors 2005; Panhuis et al. 2005), and heritability for male harm has been documented in the seed beetle *Callosobrachus maculates* (Gay et al. 2011). Thus, it is possible that the level of sexual conflict in the FEJs has settled down at a lower level of antagonism as a result of male-female coevolution, perhaps driven by a combination of energy requirements for early fecundity and reducing size over the course of their laboratory evolution. However, we are yet to assign a definitive cause for death of FEJ females upon mating with JB males. It is possible that the small-sized FEJ females also suffer from mechanical injury while being courted or during mating with large JB males, although we did not see any evidence of gross injury in the dead females in the cumulative postcopulation mortality assay.

We found no evidence for postzygotic RI, as hybrids between FEJ and JB populations were as viable as the JBs, and also nearly as fertile as the JBs (Figs. 5a,b, b6). The development time of the hybrids, however, was intermediate between the FEJs and JBs (Fig. 7a). Thus, despite the considerable evolutionary restructuring of most aspects of the preadult and adult life-history, and many related traits, in the FEJs (Prasad et al. 2000, 2001; Joshi et al. 2001; Prasad 2004), a restructuring that has resulted in substantially reduced preadult survivorship (Fig. 6a,b), there does not seem to be any intrinsic genetic incompatibility between the FEJ and JB development that would reduce hybrid viability. Duration of all preadult developmental stages, starting from embryonic development to pupal duration have been significantly reduced in FEJs compared to the JBs (Ghosh-Modak 2009), and there is evidence for large-scale changes in the temporal profile of gene expression during development in the FEJ populations (Satish 2010; K. M. Satish, P. Dey, and A. Joshi, unpubl. data). Despite such differences in the FEJs and JBs, the hybrids were viable and fertile, and their development time was intermediate, suggesting that the kinds of genetic difference needed to generate genomic incompatibilities may be rather more extensive than often thought to be the case, as also suggested by Rice and Hostert (1993). Our results, thus, support the widely held view that prezygotic isolation often evolves much faster than postzygotic isolation (Kilias et al. 1980; Coyne and Orr 1989, 1997; Rice and Hostert 1993; Coyne and Orr 2004; Vines and Schluter 2006).

Our study shows that long-term directional selection for rapid development has led to some degree of RI between selected populations of *D. melanogaster* and their ancestral controls, most likely a consequence of the correlated evolution of greatly reduced body size in the selected FEJ populations. In *Drosophila*, both development time and body size respond readily to various kinds of selection, and also show plastic responses to various environmental factors like temperature and crowding (reviewed by Prasad and Joshi 2003). Thus, RI mediated by changes in development time and/or body size could in principle be a reasonably common outcome of divergent ecological adaptation in this genus, suggesting that early stages of ecological speciation can be a by-product of differential life-history evolution, even in the absence of major differences in habitat or resource use between populations. The manner in which the body size differences appear to have mediated RI between fast developing FEJs and the JB controls is also interesting in that it seems to involve two complementary and asymmetric isolating mechanisms. Small FEJ males obtain few matings with large JB females, giving rise to unidirectional, premating RI mediated by mating success (sexual selection). Conversely, small FEJ females suffer greatly increased mortality following mating with large JB males, resulting in unidirectional viability-selection-driven postmating RI. This exemplifies the view that the manner in which life-history, sexual selection, and natural selection interact in the course of ecological speciation can be both subtle and complex (Ritchie 2007; Maan and Seehausen 2011).
